# Anchorage onto deciduous teeth: effectiveness of early rapid maxillary expansion in increasing dental arch dimension and improving anterior crowding

**DOI:** 10.1186/s40510-015-0093-x

**Published:** 2015-07-08

**Authors:** Sabrina Mutinelli, Mario Manfredi, Antonio Guiducci, Gloria Denotti, Mauro Cozzani

**Affiliations:** Private practice, via Brennero 260/B, 38121 Trento, Italy; Private practice, via Monte Cauriol 10, 38068 Rovereto, Italy; Private practice, viale Stazione 94, 54100 Massa, Italy; University of Cagliari Department of Pedodontics and Interceptive Orthodontics, via Ospedale 54, 09123 Cagliari, Italy; University of Cagliari School of Dental Medicine, IRCCS “G. Gaslini” and Hospital “Galliera” (Genova), Via Fontevivo 21 N, 19125 La Spezia, Italy

**Keywords:** Rapid maxillary expansion, Early mixed dentition, Dental arch changes, Irregularity index

## Abstract

**Background:**

Anchorage onto permanent dentition is a common procedure in rapid maxillary expansion. However, replacing first permanent molars with the second deciduous molars seems to be an option to reduce some negative side effects during orthodontic treatment. The purpose of this study was to evaluate the dental effect of rapid maxillary expansion with anchorage exclusively onto deciduous teeth performed in the first period of transition.

**Methods:**

Twenty patients with a lateral cross-bite treated exclusively by a Haas expander in early mixed dentition were retrospectively analyzed before treatment, at appliance removal, and at 21 months out of retention. The sagittal and transverse dimensions, together with the inter-canine arch and irregularity index, were digitally measured on scanned images of dental casts. The patients were compared with three balanced control groups (in total, 60 individuals) matched for gender. Two control groups had the same canine dental class as the treated group at T1, were in the inter-transitional period, and either had or lacked a lateral cross-bite. The last control group was comprised of adolescents in permanent dentition with a dental class I. The statistical analysis was performed by means of repeated-measures ANOVA for paired data and one-way ANOVA, the Kruskal-Wallis test, and the Mann-Whitney test for independent measures (α-level *p* < 0.05).

**Results:**

At the end of follow-up (inter-transitional period of dentition), the dental arch dimensions of treated patients were similar to those of adolescents with a dental class I and significantly wider than those of patients with a lateral cross-bite. Also, the anterior irregularity index was lower among patients who had undergone expansion treatments than in all untreated study participants.

**Conclusions:**

The Haas expander anchored to the deciduous teeth is effective in increasing the dental arch width in patients with a lateral cross-bite. The dimensions of the dental arch were modified earlier toward the values of the permanent dentition.

## Background

Rapid maxillary expansion (RME) is a widely used method to treat transverse deficiency [1], by opening the mid-palatal suture [2–4] during the period of skeletal growth [5, 6].

The Haas appliance [7] is a tooth-/tissue-borne device, anchored to the first molars and premolars by bands and to the palatal vault by an acrylic body. Some authors [8–11] have demonstrated the effectiveness of a modified Haas device, anchored onto the deciduous teeth (canines and second deciduous molars), in producing expansion of the transverse diameters of the upper arch. Moreover, there appears to be less relapse in the anterior area if the expansion is carried out in the first period of transition, that is, before the permanent lateral incisor will have fully erupted, and in the presence of a lateral cross-bite [12].

The anchorage onto deciduous teeth could be a viable option for preventing root resorption [2, 13–15], bone loss [16, 17], gingival recession [18], and white-spot lesions [19] on permanent dentition.

The purpose of the present study was to investigate the clinical relevance of changes in dental arch dimensions and irregularity produced by a Haas appliance anchored onto the deciduous dentition and to compare young treated patients with homogeneous untreated individuals.

## Methods

The study group was comprised of 20 Caucasian children (13 females and 7 males) in the first period of transition (mean age, 7 years and 1 month; SD, 11 months) with a mono- or bilateral cross-bite. Fifteen of the 20 patients had a canine class II.

The study group was selected retrospectively and sequentially in the clinical archives of two orthodontists who followed the same treatment protocol.

**Fig. 1:**
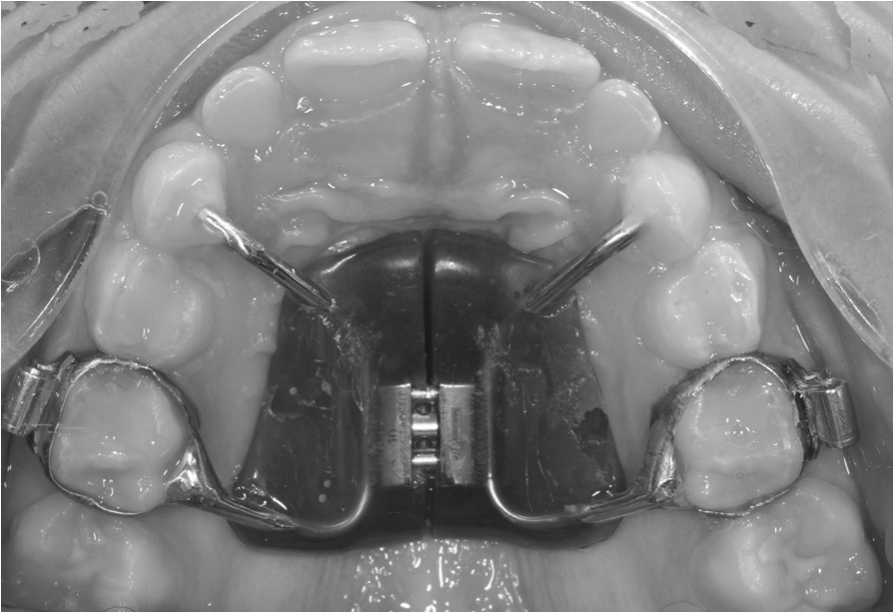
The Haas RME anchored onto the deciduous dentition

The Haas appliance was anchored to the second deciduous molars and deciduous canines (Fig. 1) and was activated once or twice per day; each activation was 0.2 mm, with a maximum allowable total expansion of 10 mm.

The patients were monitored weekly, and the expansion was terminated when the cross-bite was corrected (mean, 20 days) [9]. The appliance was kept *in situ* as retention for 12 months (SD, 4 months), after the screw was fixed.

For each patient, the dental casts before expansion (T1; mean age, 7 years and 1 month; SD, 11 months), after the appliance was removed (T2; mean age, 8 years and 1 month; SD, 10 months), and at 21 months (SD, 9 months) out of retention, during the inter-transitional period of the dentition (T3; mean age, 9 years and 10 months; SD, 1 year and 4 months), were collected.

**Fig. 2:**
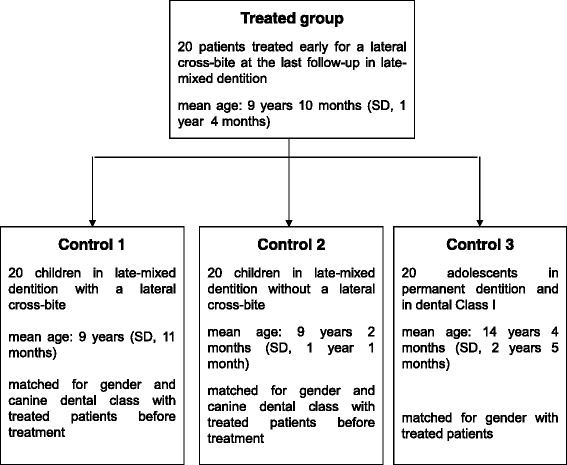
Case-control study design diagram

Three control groups were selected from among the patients sequentially checked during either the orthodontic consult or the periodic check-up for caries. For two groups, the inclusion criteria were the same canine dental class and the male-to-female ratio as the study group. The dental age coincided with the inter-transitional period [12]. Patients in one of the two groups had a lateral cross-bite (mean age, 9 years; SD, 11 months), and those in the other were without a lateral cross-bite (mean age, 9 years and 2 months; SD, 1 year and 1 month). The last control group, adolescents in permanent dentition (mean age, 14 years and 4 months; SD, 2 years and 5 months), was in normal occlusion, presented the same male-to-female ratio as the study group, and had a full natural dental arch up to the first or second molars. Impressions were taken for each patient. The case-control study design diagram is shown in Fig. 2.

The scanned images of the dental casts of all treated and non-treated individuals were measured by a computerized method described previously [20].

**Fig. 3:**
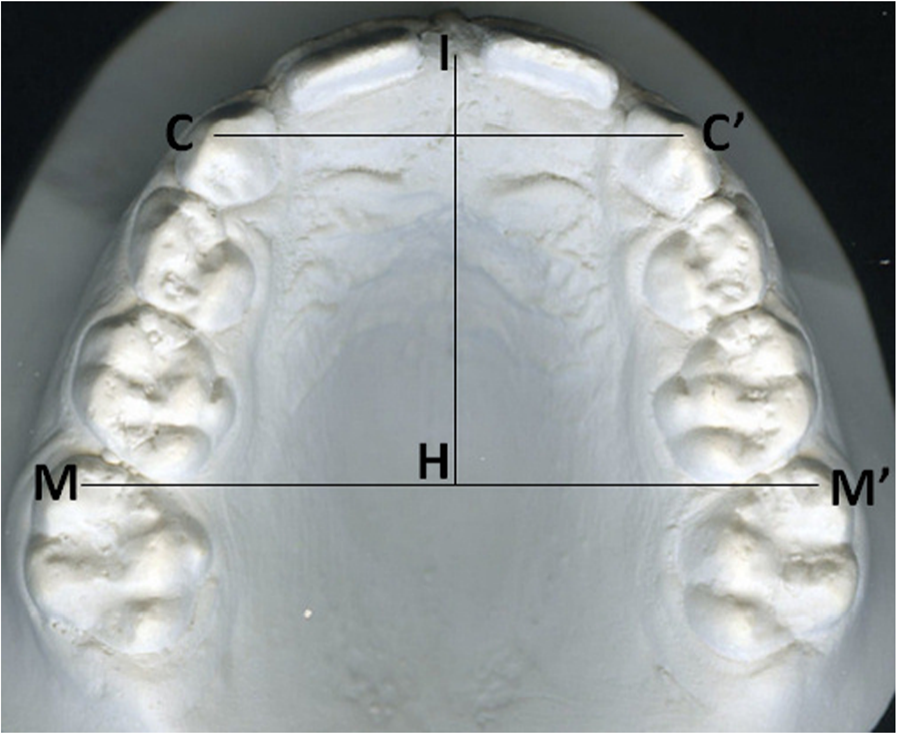
Distances drawn on an image of scanned dental cast. *C–C′* is inter-canine width, *M–M′* is inter-molar width, and *I–H* is inter-molar depth

The mesial and distal points and the tips of the cusps of each tooth and the inter-incisive point were digitally identified. If the central incisors had not erupted, the landmark of the inter-incisive point was marked with respect to the insertion of the frenulum. The measurements for each dental cast were as follows (Fig. 3):

- inter-canine width (between cusp tips of the left and right canines),
- inter-canine arch (elliptical arch between cusp tips of the left and right canines),
- inter-molar width (between mesio-buccal cusps of the left and right first molars),
- inter-molar arch depth (distance between the inter-incisive point and inter-molar width), and
- the Little irregularity index [21] applied to the upper arch.

### Statistical analysis

The normal distribution of variables was tested with a graphical visualization and the Shapiro-Wilk test. The data from the treated patients were analyzed by comparison of the values measured at T1, T2, and T3 with the repeated-measures ANOVA and the Tukey test (α level, 0.05). The null hypothesis stated that there was no difference in the dimensions of the dental arch before and after treatment.

The patients undergoing expansion were matched with the members of each control group with respect to gender for adolescents in normal occlusion and on the basis of gender and canine dental class for those in malocclusion (Fig. 2). The comparison of the measures correlated to the deciduous canines was performed only among the groups in the inter-transitional period.

The applied statistics were the one-way ANOVA and the Bonferroni test for normally distributed data and the Kruskal-Wallis equality-of-populations rank test and the Mann-Whitney test for not normally distributed variables. The null hypothesis was the equality in the dimensions of the dental arches of the treated and untreated individuals, with a significance level of *p* < 0.05.

The reliability of the operator in identifying the points on the scanned images of dental casts was calculated by the intraclass correlation coefficient (ICC). The same operator repeated the identification of landmarks of 20 patients 1 week after the first examination. The outcome was non-significant (ICC, 99.95 %), and therefore, the reliability was confirmed.

All data were statistically analyzed with STATA12 (StataCorp LP, College Station, TX, USA).

## Results

### Study group 

The statistical analysis of the variations in the study group showed, at T3, a significant increase (*p* < 0.001) in the inter-canine width (mean, 4.2 mm; SD, 2.0) and arch (mean, 6.2 mm; SD, 3.8), in the inter-molar width (mean, 4.7 mm; SD, 2.0), and in the inter-molar depth (mean, 0.8 mm; SD, 1.1). The relapse from T2 to T3 was significant (*p* < 0.001) in the inter-canine width (mean, −2.2 mm; SD, 2.0) and arch (mean, −4.1 mm; SD, 3.6) (Tables 1 and 2).

**Table 1:** Descriptive statistics and statistical analysis of the treated sample recorded at T1, T2, and T3

	Time			Comparisons		
	T1	T2	T3	T1 vs T2	T1 vs T3	T2 vs T3
	*n* = 20	*n* = 20	*n* = 20			
Inter-canine width						
mean (SD), mm	28.0(1.9)	34.4(2.4)	32.2(2.0)	S*	S*	S*
95 % CI	27.1 to 28.9	33.3 to 35.6	31.3 to 33.1			
Inter-canine arch						
mean (SD), mm	30.7(3.2)	41.1(4.7)	37.0(3.6)	*S* ^†^	*S* ^†^	*S* ^†^
95 % CI	29.2 to 32.3	38.7 to 43.3	35.3 to 38.7			
Inter-molar width						
mean (SD), mm	44.8(2.5)	49.7(1.9)	49.6(2.3)	*S* ^‡^	*S* ^‡^	*NS* ^‡^
95 % CI	43.7 to 46.0	48.7 to 50.5	48.5 to 50.6			
Inter-molar depth						
mean (SD), mm	29.8(1.7)	30.9(2.1)	30.6(1.9)	*S* ^§^	*S* ^§^	*NS* ^§^
95 % CI	29.0 to 30.6	30.0 to 31.9	29.7 to 31.4			

*S* is the significant result of the Tukey test performed after the repeated-measures ANOVA *(*F*_(2,21)_ = 17.21; *p* < 0.001; power of the test = 100 %); ^†^(*F*_(2,21)_ = 11.67; *p* < 0.001; power of the test = 100 %); ^‡^(*F*_(2,21)_ = 18.79; *p* < 0.001; power of the test = 100 %). ^§^ *S* is the significant result and *NS* is the non-significant result of the Tukey test performed after the repeated-measures ANOVA (*F*_(2,19)_ = 17.13; *p* < 0.001; power of the test = 99 %)

**Table 2:** Mean difference and 95 % CI in the measurements of the treated patients at time points T1, T2, and T3

	Differences between measurements at time-points T1, T2, and T3		
	T2–T1	T3–T1	T3–T2
	*n* = 20	*n* = 20	*n* = 20
Inter-canine width			
mean, mm	6.4*	4.2*	−2.2*
95 % CI	5.7 to 7.2	3.3 to 5.1	−3.1 to −1.3
Inter-canine arch			
mean, mm	10.3*	6.2*	−4.1*
95 % CI	8.6 to 12.1	4.4 to 8.0	−5.8 to −2.4
Inter-molar width			
mean, mm	4.8*	4.7*	−0.1
95 % CI	4.2 to 5.4	3.8 to 5.7	−0.9 to 0.7
Inter-molar depth			
mean, mm	1.1*	0.8*	−0.4
95 % CI	0.6 to 1.7	0.2 to 1.3	−0.8 to −0.04

*Significant difference

### Study group vs control groups 

#### Inter-canine width

The inter-canine width of the patients who had undergone expansion, at T3 (mean, 32.2 mm; SD, 2.0), was significantly higher than the value for children in the inter-transitional period with a lateral cross-bite (mean, 30.3 mm; SD, 3.1; *p* = 0.042) (Table 3).

**Table 3:** Descriptive statistics and statistical analysis of the treated patients at T3 and the control groups

	Groups				Comparison among groups
	Treated group	Children *with* a lateral cross-bite	Children *without* a lateral cross-bite	Adolescents with a normal occlusion	*p* value
	*n* = 20	*n* = 20	*n* = 20	*n* = 20	
Mean age (SD), years	9.8(1.3)	9.0(0.9)	9.2(1.1)	14.3(2.4)	
Inter-canine width					
mean (SD), mm	32.2(2.0)	30.3(3.1)	31.8(2.0)	-	0.0353*
95 % CI	31.3 to 33.1	28.8 to 31.7	30.9 to 32.7		
Inter-molar width					
mean (SD), mm	49.6(2.3)	44.5(2.4)	47.9(2.9)	50.6(2.8)	<0.001^†^
95 % CI	48.5 to 50.6	43.3 to 45.6	46.5 to 49.2	49.4 to 52.1	
Inter-molar depth					
mean (SD), mm	29.8(1.7)	31.8(2.2)	32.1(2.0)	29.0(1.6)	
95 % CI	29.0 to 30.6	30.8 to 32.9	31.2 to 33.0	28.3 to 29.8	<0.001^‡^
Irregularity index					
median (Iqr)^§^, mm	2.4(1.4)	3.2(2.9)	4.0(4.0)	-	0.0094^‡^

*One-way ANOVA (*F*_(2,57)_ = 3.55; power of the test = 98 %; rho = 0). Significant pair differences (Bonferroni test): treated group vs children with a lateral cross-bite (*p* = 0.042). ^†^One-way ANOVA (*F*_(3,76)_ = 21.60; power of the test = 100 %; rho = 0). Significant pair differences (Bonferroni test): treated group vs children with a lateral cross-bite (*p* < 0.001) and adolescents with a normal occlusion vs both children with a lateral cross-bite (*p* < 0.001) and children without a lateral cross-bite (*p* = 0.005). ^‡^One-way ANOVA (*F*_(3,76)_ = 12.86; power of the test = 99 %; rho = 0). Significant pair differences (Bonferroni test): treated group vs both children with a lateral cross-bite (*p* = 0.006) and children without a lateral cross-bite (*p* = 0.001); adolescents with a normal occlusion vs both children with a lateral cross-bite (*p* < 0.001) and children without a lateral cross-bite (*p* < 0.001). ^§^Iqr is interquartile range. ^ǂ^Kruskal-Wallis equality-of-populations rank test. Significant pair differences (two-sample Wilcoxon rank-sum test): treated patients vs children with a lateral cross-bite (*p* = 0.0094) and children without a lateral cross-bite (*p* = 0.0080)

#### Inter-molar width

The inter-molar width of the treated group (mean, 49.6 mm; SD, 2.3) was significantly higher than that of children with a lateral cross-bite (mean, 44.5 mm; SD, 2.4; *p* < 0.001) but was not higher than that of children without a lateral cross-bite (mean, 47.9 mm; SD, 2.9; *p* = 0.264). There was no difference between adolescents with a normal occlusion (mean, 50.6 mm; SD, 2.8) and treated patients (*p* = 0.938). Inter-molar width in adolescents was wider than that in children with (*p* < 0.001) and without a lateral cross-bite (*p* = 0.005) (Table 3).

#### Inter-molar depth

The value of the inter-molar depth was significantly lower in the treated group (mean, 29.8 mm; SD, 1.7) than in the children with (mean, 31.8 mm; SD, 2.2; *p* = 0.006) and without a lateral cross-bite (mean, 32.1 mm; SD, 2.0; *p* = 0.001). In the same manner, the difference was significant between adolescents with a normal occlusion (mean, 29.0 mm; SD, 1.6) and children with (*p* < 0.001) and without a lateral cross-bite (*p* < 0.001). There was no difference between the adolescents with a normal occlusion and the treated patients (Table 3).

#### Anterior irregularity index

The irregularity index was significantly higher in untreated children in malocclusion, whether with (median, 3.2 mm; interquartile range, 2.9; *p* = 0.0094) or without (median, 4.0 mm; interquartile range, 4.0; *p* = 0.0080) a lateral cross-bite, compared with that of the treated group (median, 2.4 mm; interquartile range, 1.4) (Table 3).

## Discussion

This research confirms the efficacy of the Haas expander anchored to the deciduous teeth in modifying the dental arch width in a group of patients with a lateral cross-bite, treated in the first period of transition. The dimension of the inter-molar width maintained a stable measure not different from that of older individuals during adolescence and in normocclusion.

In previous studies, many authors evaluated the timing of rapid maxillary expansion during growth. Sari et al. [22] found that the increase in the inter-molar and inter-canine widths was equally significant in the mixed and the permanent dentition. In contrast, Bacetti et al. [23] found that the modification in the maxillary inter-molar width was more pronounced in the patients who underwent expansion before the pubertal peak.

In the present study, the treated group was retrospectively selected following the timing for expansion reported in the paper by Mutinelli et al. [10] and corresponding to the first period of transition. The comparison at the final follow-up with three control groups allowed for evaluation of the change in treated patients with respect to that in untreated individuals. One limit of this analysis is the evaluation of the inter-canine width. The last available record for the treated patients was the dental cast in mixed dentition with the deciduous canines. Therefore, it was not possible to perform any comparison of inter-canine width between treated children in late-mixed dentition and adolescents in permanent dentition and in dental class I. Moreover, there was no follow-up of the control groups during the growth period; three groups were analyzed at two different dental ages. However, the matching of patients controlled the effects of the two well-known confounding variables, gender [24] and dental class II [25]. In addition, canine class II [26, 27] and lateral cross-bite [6] do not self-correct during dental arch growth, which is completed with the eruption of permanent canines [12, 24, 28, 29]. Therefore, it could be hypothesized that the control groups well represented the dental arch dimension of children, homologous to the treated group, at the late-mixed dentition with or without a lateral cross-bite but not treated with a rapid maxillary expansion. Further, deciding not to treat a patient with a lateral cross-bite and to follow that individual as a control is not feasible for ethical reasons. Therefore, a randomized controlled trial is difficult to implement.

The expander anchored to the deciduous teeth had, as a transverse effect, a significant increase at T3 in inter-canine width in 4.2 mm and inter-canine arch in 6.2 mm. The inter-molar diameter maintained 4.6 mm of the initial expansion (96 %) at 21 months post-retention (T3). Furthermore, the final value was no different from the values of the adolescent control group in normal occlusion. No statistically significant variation in inter-molar arch depths was recorded.

Spillane and McNamara [30] demonstrated, on average, 5 mm of residual expansion in the inter-molar width 2.4 years after expansion. Lima et al. [31] reported 4.5 mm (81.1 % of the initial expansion) 4 years after the end of activation. In an equal period of observation, this amounted to 84 % (3.7 mm) in the findings of Wong et al. [32], but these authors followed the SME and not the RME protocol of activation. The final dimension was no different from that of the control group with normal growth. At least 1 year out of retention, the residual expansion was 3.8 mm (79 %) for Mutinelli et al. [10] and 3.7 mm (90.2 %) for Cozzani et al. [9].

Schiffman and Tuncay [33] found a mean loss of 40 % of expansion in the inter-molar width within 5 years after the end of treatment. The net increase was between 3 and 4 mm in the long term, similar to the amount of normal growth in the period of time between the stages of the late-mixed and the permanent dentition. The authors did not consider that amount of increase a success, as Turpin [34] discussed in his editorial, if the aim of the treatment was to achieve a larger increase. Also, in this study, in which the global effect of the RME on the dental arch was analyzed, it can be concluded that the patients in the inter-transitional period showed sagittal and transverse dimensions no wider than those of adolescents in canine dental class I. The inter-molar arch depths were not influenced directly by the expansion, but at T3, the values were significantly lower than those in individuals at the same dental age. Normally, the upper arch modifies until the full eruption of the permanent dentition [12, 24] and has a tendency to become wider and shorter [12, 24, 35]. Further, expansion improved the transverse deficiency connected to the presence of a canine class II [27, 35]. Another important outcome was the value of the anterior irregularity index. In treated patients, it was significantly lower than in untreated individuals in malocclusion at the same dental age. Therefore, the choice to treat in the first transitional period and not later can have the goal of creating conditions conducive for development of the upper dental arch toward the dimensions of a patient in normal occlusion and not toward wider dimensions. Moreover, increasing the inter-canine arch length before the end of eruption of the permanent incisors, as reported by other authors [10, 32], can be a treatment option in middle and non-severe crowding, because the mean available space at T3 was 6.2 mm. A better alignment of the anterior teeth, a correct arrangement of the transseptal fibers, and a consequent reduction of risk in severe rotation [36] can be hypothesized. Similarly, this might confirm the results reported by Canuto et al. [37] that the RME applied later in the permanent dentition did not influence long-term maxillary anterior alignment stability.

Moreover, the option to anchor the appliance to the deciduous teeth can avoid the side effects of periodontal problems [16–18], root resorption [2, 13, 14], and white-spot lesions [19].

Further long-term studies based on a bigger sample size, more representative of the orthodontic population, are necessary to confirm these results.

## Conclusions

Rapid maxillary expansion performed in the first period of transition is effective in the treatment of patients with a lateral cross-bite. The dental arch increases in dimensions earlier toward the values of the permanent dentition stage, in comparison with dimensions in homogeneous untreated individuals. As a result, the increase in arch length can improve the alignment of the upper anterior teeth in patients with low or moderate crowding.

Therefore, early treatment with a Haas expander anchored to the primary dentition could be a satisfactory procedure for lateral cross-bite resolution and for anterior crowding improvement, due to simplicity of appliance design, ease of treatment management, and low risk of side effects.
